# Comparison of Eye Movement Desensitization Reprocessing and Cognitive Behavioral Therapy as Adjunctive Treatments for Recurrent Depression: The European Depression EMDR Network (EDEN) Randomized Controlled Trial

**DOI:** 10.3389/fpsyg.2018.00074

**Published:** 2018-02-13

**Authors:** Luca Ostacoli, Sara Carletto, Marco Cavallo, Paula Baldomir-Gago, Giorgio Di Lorenzo, Isabel Fernandez, Michael Hase, Ania Justo-Alonso, Maria Lehnung, Giuseppe Migliaretti, Francesco Oliva, Marco Pagani, Susana Recarey-Eiris, Riccardo Torta, Visal Tumani, Ana I. Gonzalez-Vazquez, Arne Hofmann

**Affiliations:** ^1^Clinical and Biological Sciences Department, University of Turin, Turin, Italy; ^2^Clinical and Oncological Psychology, Città della Salute e della Scienza Hospital of Turin, Turin, Italy; ^3^eCampus University, Novedrate, Italy; ^4^Centro INTRA-TP, A Coruña, Spain; ^5^Laboratory of Psychophysiology, Department of Systems Medicine, University of Rome “Tor Vergata”, Rome, Italy; ^6^Psychiatry and Clinical Psychology Unit, Department of Neurosciences, Fondazione Policlinico “Tor Vergata”, Rome, Italy; ^7^EMDR Italy Association, Bovisio Masciago, Italy; ^8^Center for Stress Medicine, Lüneburg, Germany; ^9^Clínica Assistens, A Coruña, Spain; ^10^Private Practice, Eckernfoerde, Germany; ^11^Department of Public Health and Pediatrics, University of Turin, Turin, Italy; ^12^Institute of Cognitive Sciences and Technologies, National Research Council, Rome, Italy; ^13^Neuroscience Department, University of Turin, Turin, Italy; ^14^Department of Psychiatry, Ulm University Hospital, Ulm, Germany; ^15^Department of Psychiatry, A Coruña University Hospital, A Coruña, Spain; ^16^EMDR Institut Deutschland, Bergisch Gladbach, Germany

**Keywords:** EMDR, CBT, depression, traumatic stress, anxiety, quality of life, antidepressants, randomized controlled trial

## Abstract

**Background:** Treatment of recurrent depressive disorders is currently only moderately successful. Increasing evidence suggests a significant relationship between adverse childhood experiences and recurrent depressive disorders, suggesting that trauma-based interventions could be useful for these patients.

**Objectives:** To investigate the efficacy of Eye Movement Desensitization and Reprocessing therapy (EMDR) in addition to antidepressant medication (ADM) in treating recurrent depression.

**Design:** A non-inferiority, single-blind, randomized clinical controlled trial comparing EMDR or CBT as adjunctive treatments to ADM. Randomization was carried out by a central computer system. Allocation was carried out by a study coordinator in each center.

**Setting:** Two psychiatric services, one in Italy and one in Spain.

**Participants:** Eighty-two patients were randomized with a 1:1 ratio to the EMDR group (*n* = 40) or CBT group (*n* = 42). Sixty-six patients, 31 in the EMDR group and 35 in the CBT group, were included in the completers analysis. **Intervention:** 15 ± 3 individual sessions of EMDR or CBT, both in addition to ADM. Participants were followed up at 6-months.

**Main outcome measure**: Rate of depressive symptoms remission in both groups, as measured by a BDI-II score <13.

**Results:** Sixty-six patients were analyzed as completers (31 EMDR vs. 35 CBT). No significant difference between the two groups was found either at the end of the interventions (71% EMDR vs. 48.7% CBT) or at the 6-month follow-up (54.8% EMDR vs. 42.9% CBT). A RM-ANOVA on BDI-II scores showed similar reductions over time in both groups [*F*(6,59) = 22.501, *p* < 0.001] and a significant interaction effect between time and group [*F*(6,59) = 3.357, *p* = 0.006], with lower BDI-II scores in the EMDR group at T1 [mean difference = –7.309 (95% CI [–12.811, –1.806]), *p =* 0.010]. The RM-ANOVA on secondary outcome measures showed similar improvement over time in both groups [*F*(14,51) = 8.202, *p* < 0.001], with no significant differences between groups [*F*(614,51) = 0.642, *p* = 0.817].

**Conclusion:** Although these results can be considered preliminary only, this study suggests that EMDR could be a viable and effective treatment for reducing depressive symptoms and improving the quality of life of patients with recurrent depression. Trial registration: ISRCTN09958202.

## Introduction

Depression is one of the most common mental disorders, affecting more than 300 million people (WHO, 2017). The consequences of this disorder in terms of health loss are huge. WHO has ranked depression as “the single largest contributor to global disability, accounting for 7.5% of all years lived with disability in 2015” (WHO, 2017).

Although over the last 20 years the options for depression therapy have increased significantly, the optimism that initially accompanied the use of new antidepressant medications (ADMs), such as selective reuptake inhibitors of serotonin (SSRIs), disappeared rapidly (Pampallona et al., 2002). In fact, several meta-analyses have concluded that ADMs have only a modest advantage over placebos (Kirsch et al., 2008; Khan and Brown, 2015), though with greater benefits in the case of severe depression (Fournier et al., 2010).

Depression treatment also involves the use of psychotherapeutic interventions, which have proved effective not only in mild and moderate depression but also in severe chronic depression (Nemeroff et al., 2003).

Guidelines indicate that for people with moderate or severe depression the most effective treatment is a combination of ADMs and a high-intensity psychological intervention (National Collaborating Centre for Mental Health [UK], 2010). Cognitive Behavioral Therapy (CBT) is one of the best known, empirically supported treatments for depression (National Collaborating Centre for Mental Health [UK], 2010). CBT is based on the premise that maladaptive cognitions contribute to the onset and maintenance of depression. According to Beck’s model, a change in these maladaptive cognitions can lead to changes in emotional regulation and dysfunctional behaviors (Beck, 1979).

In recent years, much evidence has accumulated highlighting the role of stress and its neurobiological correlates in both the occurrence and development of major psychiatric disorders, including depression (Nemeroff, 2016). The exposure to adverse childhood experiences (ACEs), which includes physical and sexual abuse as well as emotional neglect (Felitti et al., 1998; Norman et al., 2012; Infurna et al., 2016), is associated with a marked increase in the risk of developing depression in adulthood (Kendler et al., 1995; Anda et al., 2006; American Psychiatric Association, 2013; Lindert et al., 2014; Khan et al., 2015; Infurna et al., 2016; Kendler and Gardner, 2016; Nemeroff, 2016; Hughes et al., 2017).

Compared with individuals who have not experienced adverse events in childhood, those with a history of such experiences are at greater risk of having a depressive episode in their lifetime (Kessler, 1997). A graded relationship between the number of ACEs and the probability of lifetime and recent depressive disorders has also been highlighted (Chapman et al., 2004; Anda et al., 2006).

Moreover, several studies have shown that ACEs are associated with a poorer clinical course of depression, including earlier age of onset, greater severity of symptoms, co-morbidity, and episode persistence and recurrence (Heim and Nemeroff, 2001; Wiersma et al., 2009; Scott et al., 2012; Tunnard et al., 2014; Paterniti et al., 2017).

Several studies have investigated the effect of ACEs on the course of major depressive disorder (MDD), pointing out a strong association between a history of adverse events in childhood and the course of depression in adulthood (Widom et al., 2007; Infurna et al., 2016; Li et al., 2016). Also, a recent meta-analysis (Nanni et al., 2012) has suggested that childhood maltreatment is associated with an elevated risk of the recurrence and persistence of depressive symptoms. In addition, Chen J. et al. (2014) recently showed a significant association between childhood sexual abuse and recurrent major depression, with earlier age of onset and longer depressive episodes for depressed women who experienced sexual abuse in their childhood.

The clear recognition that patients with major depression who have experienced ACEs exhibit an unfavorable course of depression and a poor response to standard treatments, thereby incurring a greater risk of recurrent and persistent depressive episodes, suggests that it is essential to develop novel therapeutic approaches specifically tailored to treating traumatic experiences (Nanni et al., 2012; van Nierop et al., 2015; Nemeroff, 2016; Williams et al., 2016).

Eye Movement Desensitization and Reprocessing (EMDR) therapy was originally developed by Francine Shapiro in the late 1980s to treat traumatic memories (Shapiro, 1989). It is now widely recognized as an empirically supported treatment for post-traumatic stress disorder (PTSD) (National Collaborating Centre for Mental Health [UK], 2005; Bisson and Andrew, 2007; Chen Y.-R. et al., 2014).

EMDR therapy is guided by the Adaptive Information Processing (AIP) model (Shapiro, 2001). One of the key aspects of the AIP model is that stressful events that have not been fully processed and integrated into already existing memory networks are stored in a dysfunctional way. These stressful events do not necessarily fulfill Criterion A for PTSD and are the basis of several mental disorders, including PTSD, affective disorders, chronic pain, and addiction (Shapiro, 2014; Hase et al., 2017). A recent study (Hase et al., 2017) proposed a link between dysfunctionally stored memory and the theory of pathogenic memory, previously described by Centonze et al. (2005).

The reactivation of a pathogenic memory induced by various internal and external stimuli, also exerting vegetative arousal, could lead to subsequent maladaptive responses, which in the long-term could contribute to the onset of various psychiatric disorders (Hase et al., 2017). From this perspective, it could be hypothesized that pathogenic memories contribute to the onset and maintenance of recurrent depression episodes. By promoting the reprocessing of pathogenic memories, EMDR may represent a promising approach and thus could broaden the range of effective interventions for this disorder.

In recent years, the application of EMDR beyond PTSD has expanded rapidly. It is currently being used as a treatment for a wide range of disorders that follow distressing life experiences (Shapiro and Maxfield, 2002). Several books, conference presentations, and case reports suggest its applicability in treating depression too (Wood and Ricketts, 2013; Luber, 2016).

Two studies reviewing the literature on the application of EMDR to depression as primary diagnosis concluded that EMDR showed preliminary promise as a therapy for treating this disorder, although further research was required (Wood and Ricketts, 2013; Valiente-Gómez et al., 2017).

More recently, other studies have reported evidence of EMDR efficacy in patients with depression (Hofmann et al., 2014; Behnammoghadam et al., 2015; Hase et al., 2015; Mauna Gauhar, 2016), while a specific EMDR therapy protocol for the treatment of depressive disorders has been published (Hofmann et al., 2016). Moreover, a recently published study has shown the feasibility of using EMDR treatment in patients with recurrent and/or long-term depression (Wood et al., 2017).

In 2010, a group of European researchers founded the European Depression EMDR Network (EDEN) with the purpose of evaluating the efficacy of EMDR in this disorder in different contexts and with different methodologies. The underlying hypothesis is that EMDR therapy could directly address memories of adverse and traumatic experiences that are significant contributors to the onset and maintenance of depressive episodes.

The present study represents one of the Network’s research projects, its aim being to assess whether patients with recurrent depressive disorders benefit from a trauma-adapted psychotherapeutic intervention (EMDR) compared with a more classical intervention (CBT), in addition to standard clinical management and medication.

The primary aim of the study was to evaluate the efficacy of EMDR compared with CBT in terms of response rates and time frame of depressive symptoms remissions. A secondary aim was to compare the efficacy of both treatments on associated symptoms and quality of life.

## Materials and Methods

### Design

This study was a non-inferiority, randomized controlled clinical trial investigating the efficacy of EMDR treatment compared with CBT intervention in patients with recurrent depressive disorder already undergoing “treatment as usual” (TAU).

The study is registered in the ISRNCTN registry as ISRCTN09958202.

### Setting

The study was a multicenter trial, and therefore patients were consecutively recruited between 2014 and 2016 from two settings: in Italy, participants were recruited from the psychiatric services affiliated with the University Hospital San Luigi Gonzaga of Orbassano, Turin; in Spain, patients were enrolled at the Assistens Clinic, A Coruña.

This study was approved by the Research Ethics Committee of the University Hospital San Luigi Gonzaga and by the Ethical Committee of Clinical Research of Galicia. Informed written consent was obtained from all participants.

### Participants

The participants in the study consisted of 82 patients with recurrent depressive episodes, who had been referred to one of the two above-mentioned specialized clinical services and were already receiving TAU (ADMs and psychiatric visits, with stabilized ADMs for at least four weeks).

Participants were pre-screened using the Beck Depression Inventory-II (BDI-II; Beck and Steer, 1993) during a routine clinical visit. Those with a score on BDI-II greater than 13 (considered the clinical cut-off for screening of depression symptoms) were assessed using the Mini-International Neuropsychiatric Interview-Plus (MINI-Plus; Sheehan et al., 1998) clinical interview, in order to confirm the diagnosis.

Inclusion criteria were as follows: (1) a diagnosis of recurrent depressive disorder (F33.x or F33.x + F34.1 “double depression”)— this could be chronic depression (of at least two years’ duration); (2) aged between 18 and 65 years; (3) a score of at least 13 on Beck’s Depression Inventory-II (BDI-II); (4) having received ADM treatment for at least four weeks; (5) legal capacity to consent to the treatment.

Exclusion criteria were as follows: (1) a history of psychotic symptoms or schizophrenia; (2) bipolar disorder or dementia; (3) cluster A and B severe personality disorders; (4) dissociative disorders (DES score >25%); (5) any substance-related abuse or dependence disorder (except those involving nicotine) in the 6 months prior to the study; (6) a serious, unstable medical condition; (7) being pregnant; (8) undergoing parallel legal processes or applications for pension or social security.

### Recruitment and Measures

The recruitment of participants was carried out by psychiatrists, who proposed their participation in the research protocol to patients during a routine clinical visit.

The research protocol and aims of the study were explained to patients who met the inclusion/exclusion criteria. They were also told that if they took part in the study they would be randomly assigned to one of two treatment conditions, both employing the same timing and assessment tools, for the period of the study. If they agreed they signed the informed consent, were randomized, and then asked to proceed with the psychological assessment.

The following psychological self-report questionnaires were administered:

#### Beck Depression Inventory-II (BDI) (Beck and Steer, 1993)

This is a 21-item self-report instrument that assesses the presence and severity of depressive symptoms, based on DSM-IV criteria. The total score ranges from 0 to 63, with higher scores indicating higher levels of depression. A score greater than 13 is considered the cut-off for the presence of depressive symptoms (14-19: mild depression; 20-28: moderate depression; ≥29: severe depression).

#### Beck Anxiety Inventory (BAI) (Beck and Steer, 2013)

This is a 21-item self-report measure that assesses cognitive, somatic, and affective anxiety symptom severity. The total score ranges from 0 to 63, with higher scores indicating higher levels of anxiety. A score above 9 suggests the presence of clinical anxiety (10-16: mild anxiety; 17-29: moderate anxiety; ≥30: severe anxiety).

#### Impact of Event Scale-Revised (IES-R) (Weiss and Marmar, 1997)

The IES-R is a 22-item self-report questionnaire consisting of three subscales (eight items relate to intrusions, eight items evaluate avoidance, and six items assess hyperarousal). The overall scale assesses subjective distress caused by traumatic events.

#### WHO-Quality of Life Bref (WHOQOL-Bref) (Murphy et al., 2000)

The WHOQOL-Bref consists of 26 items that measure the following broad domains: physical health (WHO-Phys); psychological health (WHO-Psychol); social relationships (WHO-Social); and environment (WHO-Env).

#### Global Assessment of Functioning Scale (GAF) (American Psychiatric Association, 2000)

This scale is included in the V Axis of DSM-IV and is used by mental health providers to rate patients’ social, occupational, and psychological functioning. Scores range from 100 (extremely high functioning) to 1 (severely impaired).

The following tools were administered at the beginning of the study only:

#### The Dissociative Experiences Scale (DES) (Bernstein and Putnam, 1986; Frischholz et al., 1990)

It is a brief, 28-item self-report inventory of the frequency of dissociative experiences. It is a reliable and valid measure for determining the contribution of dissociation to various psychiatric disorders and a screening instrument for dissociative disorders. In this study, a score above 25 was considered an exclusion criterion.

#### The Trauma Antecedent Questionnaire (TAQ) (Luxenberg et al., 2001)

It is a self-administered instrument that gathers information about ACEs and other life experiences, assessed at four different age periods: early childhood (birth to 6 years), latency (7 to 12 years), adolescence (13 to 18 years), and adulthood. For each item of the TAQ, respondents are asked to rate the extent to which they have had a particular experience during each developmental period on a scale from 0 to 3. Presence of ACE is calculated when at least one adverse experience of an intensity of at least 2 is reported.

### Randomization and Assessment Points

Patients were randomly allocated to one of the two conditions: TAU+EMDR or TAU+CBT. Patients were randomized at a 1:1 ratio, using a block-wise randomization sequence (block size of four). The sequence was determined by an independent statistical consultant, blind to the initial assessments in order to ensure that allocation remained unknown, using a centralized randomization algorithm.

In each center, treatment allocation was communicated to the patients by the study coordinator to ensure that evaluators remained blind to their allocation.

The psychological assessment was performed by psychologists independent of the research protocol, using the same tools and at the same time periods for both groups: at baseline (T0), at the end of the treatment (T1), and 6 months after the end of the treatment (T2).

In order to assess the trend of depressive symptoms, four clinical management visits were also scheduled for each patient during the treatment phase. The first assessment (Assess-1) was scheduled after the first two treatment sessions, and each successive assessment (Assess-2, Assess-3, and Assess-4) was conducted every four treatment sessions. During these intra-treatment assessments, psychiatrists independent of the research protocol administered the Beck Depression Inventory-II only.

### Interventions

The clinical psychologists conducting the clinical assessments were both independent and blind to the interventions.

All patients in the study continued to receive Treatment as Usual, which comprised ADMs and the clinical management provided by each center.

The number of adjunctive EMDR or CBT individual sessions was allowed to vary between 12 and 18 (15 ± 3). This relatively flexible range of sessions was chosen with a twofold aim: (1) to avoid any large disparity in treatment between patients and centers, as no therapist would be allowed to schedule a number of sessions <12, or >18; (2) to allow therapists to schedule the appropriate number of sessions for each patient, albeit within the defined range, according to patients’ needs.

The sessions were scheduled on a weekly basis where possible. The duration of the intervention depended mainly on the number of sessions completed by each patient. Overall, it varied from between three and 6 months (e.g., when a period of vacation interrupted the treatment phase or logistical difficulties made it difficult for a patient to maintain a weekly schedule).

#### Eye Movement Desensitization and Reprocessing

The EMDR treatment followed the DeprEnd protocol; that is, the manual for EMDR in the treatment of depressive patients (see Hofmann et al., 2016 for a detailed explanation).

Eye Movement Desensitization and Reprocessing therapy intervention started with a stabilization phase consisting of two stages: in the first two sessions, the Safe Place procedure (Shapiro, 2001) and the Absorption technique (Hofmann, 2009) were used. The second phase, lasting for the following three sessions, was based on Self-care procedures (Gonzalez-Vázquez and Mosquera-Barral, 2012).

The remaining sessions focused on trauma reprocessing. EMDR targets were selected taking into account four factors that play a major role in the emergence, maintenance, and recurrence of depressive episodes. Depending on the individual life history of the patient, one or all of the following forms of pathogenic memory networks became a focus of EMDR treatment:

(1) Episode triggers of the current depressive episode (and earlier episodes): when depressive episodes appear to be triggered for the most part by either traumatic (PTSD Criterion A) or non-traumatic (not fulfilling Criterion A) events;
(2) Belief systems: when a patient undergoes a series of repeated experiences (mostly non-Criterion A events, like humiliation) that become crystallized in the form of belief systems, increasing vulnerability and the maintenance of depressive episodes;
(3) Depressive states: when patients experience earlier, longer, more intense, or repeated depressive episodes that can be remembered in a state-specific way;
(4) Depressive and suicidal states: when the memory of depression and/or suicidality itself (or suicide attempts) has created a memory structure of its own.

The EMDR targets were prioritized according to the clinical state of the patient.

In each center, EMDR was provided by three psychotherapists specializing in Level II EMDR and with a minimum of three years of experience in treating patients with depression. They received extensive training and supervision in the manualized protocol established for the study, from a certified senior EMDR instructor.

#### Cognitive Behavioral Treatment

The CBT treatment followed the manual of cognitive therapy for depression (Beck, 1979). The therapy works systematically with dysfunctional beliefs and teaches self-monitoring of negative emotions and their influence on behaviors. In addition, it includes decision-making training and targeted work on how to increase the frequency and quality of pleasant experiences. Homework assignments help patients to improve social skills in their everyday life.

In each center, CBT treatment was performed by three psychotherapists with certified training in CBT techniques and a minimum of three years’ experience in treating patients with depression. They received regular CBT supervision to ensure that the quality of their CBT treatment was maintained.

### Sample Size

Given the trial’s non-inferiority design [Null hypothesis H_0_: π_2-_π_1_ ≤–0,2 (non-inferiority)], sample size estimation was based on the formula of Farrington and Manning, the maximum likelihood method (Farrington and Manning, 1990), and implemented by ADDPLAN 4.0.3 software [Adaptive Design and Analyses, ADDPLAN 4.0.3. ADDPLAN GmbH, 2002 Cologne].

In the analysis, a single stage (fixed sample size) design and an allocation ratio (n_2_/n_1_) = 1 were considered.

For specified α = 0.05, rates π_1_ = 0.3, and π_2_ = 0.4 (odds ratio of 1.556), 62 patients (31 per group) were needed to reach a power (1–β) equal to 80.0%. In order to take 25% of dropouts and loss to follow up into account, we planned to include a total number of 82 patients.

### Statistical Analyses

Data were processed and analyzed using the Statistical Package for Social Sciences (SPSS version 22.0; Chicago, IL, United States).

Both parametric and non-parametric tests were used, in accordance with Shapiro–Wilk, as a test for normality. Baseline group differences were assessed using Student’s *t*-test or Mann–Whitney *U* test to compare the two groups on continuous measures, and Fisher’s Exact Test for categorical measures.

The primary outcome of the study was the rate of depressive symptoms remission in both groups, as measured by a BDI-II score <13. Based on the BDI-II score, patients were classified as either asymptomatic or symptomatic (BDI-II score <13/≥13, respectively) and with or without symptoms remission (BDI-II score <9/≥9, respectively), while the difference between the EMDR and CBT groups at T1 and T2 was analyzed using Fisher’s Exact Test.

Another primary aim was to compare the time frame of depressive symptoms reduction in the two groups. A GLM repeated measures ANOVA (RM-ANOVA) was used to analyze the effects of time and the interaction between time and groups (EMDR vs. CBT) for BDI-II levels across the multiple assessment points.

A secondary outcome of the study was to compare the efficacy of both treatments on associated symptoms and quality of life. A GLM repeated measures multivariate ANOVA (RM-MANOVA) was used to analyze the main pre- and post- intervention effects and interactions both between and within EMDR and CBT groups for the other clinical variables (BAI, IES-Total, WHO, GAF).

The results are shown as F (ʋ1, ʋ2), with ʋ1 and ʋ2 as numerator and denominator degrees of freedom, respectively.

Pairwise comparison between both groups and times was achieved by simple contrast and reported as means difference with Sidak correction 95% Confidence Interval (95%CI) for multiple comparisons.

Finally, an exploratory intention-to-treat analysis (ITT) was performed on the primary outcome only (i.e., BDI-II scores), with missing data accounted for using Multiple Imputation models (Howell, 2008).

A *p* < 0.05 was considered statistically significant for all the analyses.

## Results

**FIGURE 1:**
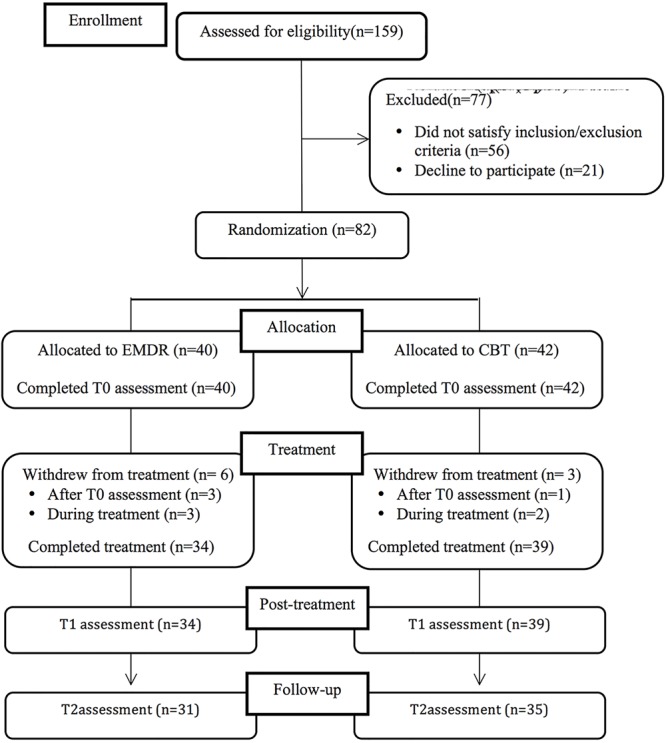
Participants flow diagram.

**Figure 1** shows a flow diagram with the number of participants at each assessment stage. A total of 159 patients were screened using the BDI-II; 56 patients were excluded on the basis of the inclusion/exclusion criteria (35.2%); and 21 refused to participate (refusal rate: 20.4%); reasons given for refusal were mainly the distance of patients’ place of residence from the place of treatment and the inability to attend the psychiatric and psychotherapeutic sessions). Eighty-two patients were randomized: 40 were assigned to the EMDR intervention and 42 to the CBT intervention. Four patients did not begin the treatment (three in the EMDR group and one in the CBT group), and five patients (three in the EMDR group and two in the CBT group) attended fewer than half of the treatment sessions. These patients refused to continue with the assessment at post-treatment and follow-up assessments and therefore it was not possible to include them in the statistical analysis. Moreover, seven patients were lost to the follow-up evaluation.

Therefore, a total of 66 patients (31 in the EMDR group and 35 in the CBT group) were included in the per-protocol statistical analysis.

**Table 1** presents the socio-demographic and clinical characteristics of these patients at baseline. There were no significant differences in demographics or clinical characteristics between the two groups at baseline (T0). In particular, both groups reported a high proportion of ACEs in the 0-18 years age period (96.7% in the EMDR group and 94.3% in the CBT group; *p* = 1.000). At the same time, no patient reported any co-morbidity with PTSD, as assessed by the MINI-Plus clinical interview at baseline.

**Table 1:** Demographic and clinical data of participants at baseline.

	EMDR (*N* = 31) Mean (SD)/Median (IQR)	CBT (*N* = 35) Mean (SD)/Median (IQR)	*p*
Age (years)	48.23 (9.66)	47.54 (12.90)	0.810^a^
Education (years)	13.00 (6.3)	12.00 (7)	0.446^b^
Age onset depression diagnosis	24.50 (21.3)	28.00 (24.5)	0.382^b^
DES	11.00 (12)	9.00 (13.5)	0.113^b^
		
	***n*(%)**	***n*(%)**	
		
Gender			0.290^c^
Female	25 (80.65)	31 (88.57)	
Male	6 (19.35)	4 (11.43)	
Employment status			0.505^c^
Unemployed	5 (16.13)	4 (11.43)	
Employed	22 (70.97)	24 (68.57)	
Pensioned	4 (12.90)	6 (17.14)	
Student	0 (0)	1 (2.86)	
Marital status			0.893^c^
Single	9 (29.03)	8 (22.86)	
Married/Cohabitee	20 (64.52)	25 (71.42)	
Separated/divorced	1 (3.225)	1 (2.86)	
Widowed	1 (3.225)	1 (2.86)
Depression diagnosis			0.706^c^
Chronic depressive disorder	3 (9.675)	6 (17.15)	
Double depression	3 (9.675)	4 (11.43)	
Recurrent depressive disorder	25 (80.65)	25 (71.42)	
TAQ			
0-6	21 (67.74)	25 (71.43)	0.793^c^
7-12	28 (90.32)	30 (85.71)	0.713^c^
13-18	30 (96.77)	33 (94.28)	1.000^c^
Adult	31 (100)	35 (100)	–

*EMDR, Eye Movement Desensitization and Reprocessing group; CBT, Cognitive Behavioral Therapy group; DES, Dissociative Experience Scale; TAQ, Trauma Antecedent Questionnaire. ^*a*^Pearson’s independent samples t-test. ^*b*^Mann-Whitney U test. ^*c*^Fisher’s exact test.*

The number of individual treatment sessions was similar for both groups (EMDR: *M* = 15.1, *SD* = 1.11; CBT: *M* = 14.6, *SD* = 1.77; *p* = 0.209).

First, for our primary outcome measure we examined the proportion of patients who no longer had a BDI-II score above the cut-off (i.e., BDI-II score > 13) at the end of the treatment (T1) and at follow-up assessment (T2). At T1 we found that 22 out of 31 patients (71.0%) in the EMDR group and 17 out of 35 patients (48.7%) in the CBT group did not have a score above the clinical cut-off for depression. At T2 we found that 17 out of 31 (54.8%) in the EMDR group and 15 out of 35 patients (42.9%) in the CBT group did not have a BDI-II score above the clinical cut-off. No significant difference between the two groups was found at either T1 or T2.

We also examined the proportion of patients who recorded a BDI-II score below 9, which is considered the clinical threshold for complete symptoms remission. At T1, 18 out of 31 patients (58.1%) in the EMDR group and 11 out of 35 patients (31.4%) in the CBT group had a BDI-II score <9, with a statistically significant difference in favor of the EMDR group (χ^2^= 4.735, *p* = 0.046). At T2 we found that 13 out of 31 patients (41.9%) in the EMDR group and 13 out of 35 patients (37.1%) in the CBT group had a BDI-II score below 9, with no significant difference between the two groups.

**FIGURE 2:**
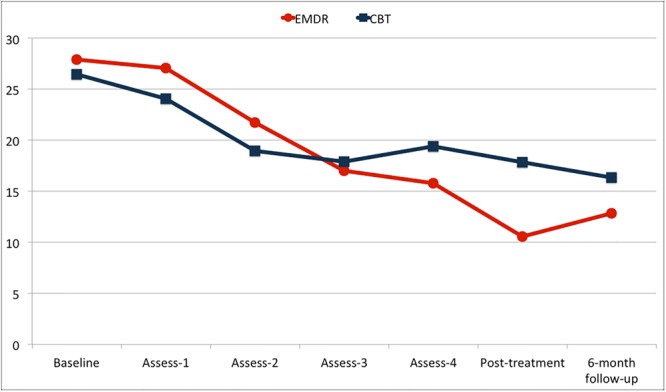
Trend of BDI-II scores for the two groups [Eye Movement Desensitization and Reprocessing group (EMDR) and Cognitive Behavioral Therapy group (CBT)].

We then investigated whether the different psychotherapy treatments (EMDR or CBT) had a different impact on BDI-II trend over time. A repeated-measures ANOVA was performed comparing group and time effects as well as interactions between group and time for BDI-II scores across the seven assessment points (i.e., baseline, four assessments during treatments, post-treatment, and 6-month follow-up). Descriptive scores are shown in **Figure 2**. The RM-ANOVA yielded a significant time main effect [*F*(6,59) = 22.501, *p* < 0.001], showing significantly reduced BDI-II scores over time for both groups. The RM-ANOVA also revealed a significant interaction effect between time and group [*F*(6,59) = 3.357, *p* = 0.006]. Planned *post hoc* analyses of simple effects with Sidak correction showed a significant difference between the two groups at post-treatment (T1), with lower BDI-II scores in the EMDR group (*M* = 10.55, *SE* = 2.006) compared with those in the CBT group (*M* = 17.86, *SE* = 1.888), with mean difference = –7.309 (95% CI [–12.811, –1.806]), *p =* 0.010 (**Figure 2**). *Post hoc* analysis of simple effects also showed a similar trend of reduction in both groups until Assessement-2, with both showing a significant difference between baseline and Assessment-2 (EMDR: mean difference = 6.161 (95%CI [1.186, 11.136]), *p* = 0.005; CBT: mean difference = 7.543 (95%CI [2.861, 12.225]), *p* < 0.001). Thereafter, the trends of the two groups differed: the CBT group showed no statistically significant difference between Assessment-2 and post-treatment (T1), mean difference = 1.806 (95%CI [–4.159, 6.331]), *p* = 1.000, while in the EMDR group there were a significant reduction in BDI-II scores between Assessment-2 and post-treatment (T1), mean difference = 11.194 (95%CI [5.620, 16.767]), *p* < 0.001 (**Figure 2**).

An ITT analysis based on Multiple Imputation models of BDI-II trend over time was also performed on the whole randomized sample, confirming the finding obtained in the completers analysis of a significant difference between EMDR and CBT at T1 (*p* = 0.011).

Moreover, for our secondary outcome we examined whether the different psychotherapy treatments (EMDR or CBT) administered to the patients had a different impact on psychological variables relating to depression. A repeated-measures MANOVA was performed on baseline, post-treatment, and follow-up secondary outcome scores (i.e., BAI, IES-R, WHO-Phys, WHO-Psychol, WHO-Social, WHO-Env, GAF), comparing group and time effects as well as interactions between group and time. This analysis yielded a significant time main effect [*F*(14,51) = 8.202, *p* < 0.001], while no significant interaction was found between time and group [*F*(614,51) = 0.642, *p* = 0.817]. The mean participant scores of all secondary outcome variables improved from baseline (T0) to post-treatment (T1) and follow-up evaluation (T2), without significant differences between the groups (**Table 2**).

**Table 2:** Comparison of clinical variables between T0, T1, and T2 for the two groups (EMDR and CBT).

	Pre-treatment (T0)		Post-treatment (T1)		6 month follow-up (T2)		Time effect^∗^
	*EMDR* (*N* = 31)	*CBT* (*N* = 35)	*EMDR* (*N* = 31)	*CBT* (*N* = 35)	*EMDR* (*N* = 31)	*CBT* (*N* = 35)	
BAI	23.23 (10.77)	27.94 (13.69)	13.55 (10.47)	19.03 (12.80)	12.61 (9.82)	17.80 (13.55)	*F*(2,128) = 33.549, *p* < 0.001; $\eta_{p}^{2}$ = 0.344
IES-R	39.29 (20.74)	37.49 (23.39)	23.00 (21.81)	26.97 (22.77)	20.23 (17.92)	24.49 (21.88)	*F*(2,128) = 27.421, *p* < 0.001; $\eta_{p}^{2}$ = 0.300
WHO-Phys	11.34 (2.31)	11.92 (2.32)	13.05 (2.28)	13.08 (2.53)	13.27 (2.10)	13.31 (2.79)	*F*(2,128) = 13.457, *p* < 0.001; $\eta_{p}^{2}$ = 0.174
WHO-Psychol	9.53 (1.83)	9.24 (1.46)	12.02 (2.25)	10.69 (2.54)	11.99 (2.47)	11.05 (2.50)	*F*(2,128) = 28.945, *p* < 0.001; $\eta_{p}^{2}$ = 0.311
WHO-Social	10.92 (2.52)	11.16 (2.46)	12.60 (2.38)	11.70 (2.19)	12.73 (2.62)	12.53 (3.09)	*F*(2,128) = 9.395, *p* < 0.001; $\eta_{p}^{2}$ = 0.128
WHO-Env	12.37 (2.11)	12.26 (2.20)	13.42 (1.74)	12.74 (2.12)	13.29 (1.77)	13.09 (2.29)	*F*(2,128) = 8.405, *p* < 0.001; $\eta_{p}^{2}$ = 0.116
GAF	68.10 (11.90)	63.66 (16.93)	77.90 (10.97)	74.60 (17.84)	77.87 (13.09)	74.94 (10.72)	*F*(2,128) = 23.557, *p* < 0.001; $\eta_{p}^{2}$ = 0.269

*Data are mean (SD). EMDR, Eye Movement Desensitization and Reprocessing group; CBT, Cognitive Behavioral Therapy group; BAI, Beck Anxiety Inventory; IES-R, Impact of Event Scale-Revised; WHO-Phys, WHO-Quality of Life Bref-Physical health; WHO-Psychol, WHO-Quality of Life Bref-Psychological health; WHO-Social, WHO-Quality of Life Bref-Social relationships; WHO-Env, WHO-Quality of Life Bref-Environment; GAF, Global Assessment of Functioning scale. ^∗^Significant time effect, independent of the type of treatment (EMDR or CBT)*.

Planned *post hoc* analysis using Sidak correction showed that in the EMDR group all the clinical scores showed improvement both between T0 and T1 and between T0 and T2, while in the CBT group similar improvement was observed for all variables except WHO-Social and WHO-Env, which showed significant improvement between T0 and T2 but not between T0 and T1 (**Table 2**).

## Discussion

Depression is the condition considered to bear the greatest responsibility for health decrements worldwide, due to its prevalence and its chronic and recurrent nature (WHO, 2017). Therefore, understanding its etiology and identifying effective and lasting treatments is a global health priority.

Antidepressant medication are the current standard of treatment in clinical practice, but they appear to be symptom-suppressive rather than curative (Hollon et al., 2002) and do not appear to maintain their effectiveness in terms of reducing future risk of depressive episodes once their course is completed (DeRubeis et al., 2008).

Therefore, identifying additional interventions that are effective in treating depression and reducing the risk of its recurrence to lasting effect, is of the utmost importance.

To the best of our knowledge, this is the first randomized controlled trial to evaluate the efficacy of EMDR in comparison with CBT in patients affected by recurrent depression and treated with ADM.

The most significant result highlighted by this study is that the majority of patients were able to significantly reduce their depression symptoms level after only 15 therapy sessions, and to sustain this clinical benefit 6 months after the end of the psychotherapeutic intervention.

Eye Movement Desensitization and Reprocessing therapy treatment was shown to be as effective as CBT in reducing the proportion of patients with a level of depressive symptoms above the clinical threshold, both at the end of the treatment and 6 months later, with response rates similar to those reported in previous studies (DeRubeis et al., 2005; Hollon et al., 2005).

At the same time, EMDR exceeded CBT in terms of the proportion of patients who could be considered to be in remission after the end of the interventions. In addition, the results for depressive symptoms trend showed that both interventions were effective in reducing clinical levels of depression, with a significant difference in favor of EMDR treatment at the end of the intervention phase. This difference was no longer present at the 6-month follow-up, although in the EMDR group there was a tendency to remain below the clinical threshold that was not apparent in the CBT group.

Interestingly, EMDR and CBT showed a similar trend of clinical improvement in depressive symptoms in the initial phase of the intervention (i.e., until Assessment-2), but then exhibited different trajectories between Assessment-2 and post-treatment (T1). In this second phase, EMDR continued to significantly reduce depression levels until the end of the intervention, while CBT only maintained the gains made in the first phase. It is possible to interpret this result by looking in-depth at the contents of the treatment sessions. In the first four to five sessions, EMDR treatment focused on assessment and stabilization, thus exerting a similar effect to that of CBT. After EMDR’s specific work on trauma reprocessing started (around Assessment-3), EMDR showed an increase in effectiveness while CBT effects remained virtually unchanged.

The upturn in depression levels recorded at follow-up in the EMDR group may have been due to the low volume of EMDR provided. It might be hypothesized that a greater number of EMDR sessions would have facilitated more reprocessing of the pathogenic memories underlying depressive symptoms and thus the upturn could have been prevented.

As regards the secondary outcome of the study, both treatments were effective in reducing anxiety and post-traumatic symptoms even after just a limited number of sessions, with the benefits still apparent 6 months after the end of the psychological treatment. EMDR and CBT have both been proven to be efficacious in treating anxiety and post-traumatic symptoms, and therefore these results are in agreement with previous literature (Kar, 2011; Hofmann et al., 2012; Chen Y.-R. et al., 2014).

Furthermore, both treatments were able to significantly improve Quality of Life (QoL) and global functioning, the benefits here too persisting beyond the end of the intervention. The benefits associated with social and environmental QoL appeared to became apparent faster for the EMDR group, which also showed considerable improvement in these variables at the end of therapy, while the CBT group appeared to gain these benefits at a later stage. This difference could be due to the different focus of the two psychotherapeutic interventions; while CBT focuses mainly on maladaptive beliefs underlying depression, in EMDR therapy the reprocessing of dysfunctionally stored memories can lead to changes in different symptoms or in the impairment of functioning connected to the reprocessed memory, as proposed in the AIP-Model of EMDR therapy.

Moreover, the majority of patients in our study reported previous adverse childhood experiences and stressful life events (e.g., sexual and physical abuse, traumatic mourning, abandonment, and serious neglect). This finding is in line with the hypothesis that stressful life events play a significant role in both the onset and the risk of recurrence of depressive episodes (Chapman et al., 2004; Nanni et al., 2012; Pietrek et al., 2013; Nemeroff, 2016).

This study has a number of strengths. It is the first study to compare the efficacy of EMDR with that of CBT for patients with depressive disorder treated with ADMs using a randomized controlled design and evaluating the effects on associated symptoms and QoL.

### Limitations

The number of patients treated with EMDR and CBT included in the study is not large. As this is the first study attempting to investigate the non-inferiority of EMDR compared with CBT, it is possible that actual differences between the two groups were not revealed due to the design and sample size of the study; future superiority clinical trials are needed to broaden this investigation. Moreover, in this study a self-report measure (BDI-II) was used as the primary outcome measure. Future studies should also include a clinician report measure administered by an independent rater in order to overcome this limitation.

Another limitation is that the 6-month follow-up evaluation was not long enough to examine the recurrence rate of subsequent depressive episodes. Therefore, longer follow-ups (e.g., at 1 year or longer) are needed in order to identify possible differences between the two interventions in reducing the risk of recurrence of depressive episodes. Lastly, another limitation of this study was the inclusion of ITT analysis for the primary outcome only.

Although our results can only be considered preliminary, this study suggests that EMDR could be as effective as CBT in reducing depressive symptoms in patients suffering from recurrent depressive disorder and treated with ADMs. Both EMDR and CBT as adjunctive interventions to ADMs are effective in reducing anxiety and post-traumatic symptoms and increasing QoL, even over a limited number of treatment sessions.

## Author Contributions

AH, LO, MC, IF, MH, and AG-V were responsible for the conception and design of the study. MC, PB-G, AJ-A, ML, FO, SR-E, and VT were responsible for data collection. SC, GM, and FO were responsible for the data analysis. MP, RT, and GDL contributed to the interpretation of data. LO and SC wrote the article, which was critically revised by all the others authors. All authors approved the final version of the manuscript.

## Conflict of Interest Statement

IF is the president of EMDR Europe Association and EMDR Italy Association. AH is the director of EMDR Institute Germany, which conducts research and teaches in the field of EMDR. LO, MH, AG-V, IF, and AH are EMDR supervisors. LO, SC, MH, ML, MP, VT, AG-V, and AH have been invited speakers at national and international EMDR conferences. The other authors declare that the research was conducted in the absence of any commercial or financial relationships that could be construed as a potential conflict of interest. The handling Editor declared a shared affiliation, though no other collaboration, with one of the authors, MC.
